# Airway Management in the ICU and Emergency Department in Resource-Limited Settings

**DOI:** 10.3390/life16020195

**Published:** 2026-01-23

**Authors:** Sahil Kataria, Deven Juneja, Ravi Jain, Tonny Veenith, Prashant Nasa

**Affiliations:** 1 Department of Critical Care Medicine, Holy Family Hospital, New Delhi 110025, India; sahilkataria2612@gmail.com; 2 Institute of Critical Care Medicine, Max Super Specialty Hospital Saket, New Delhi 110017, India; 3 Department of Critical Care Medicine, Mahatma Gandhi Medical College and Hospital, Jaipur 302022, India; ravijainstar@gmail.com; 4 Department of Critical Care Medicine and Anaesthesia, The Royal Wolverhampton NHS Trust, New Cross Hospital, Wolverhampton WV10 0QP, UK; tonny.veenith1@nhs.net; 5 Institute of Acute Care, The Royal Wolverhampton NHS Trust, University of Wolverhampton, Wolverhampton WV10 0QP, UK; 6 Department of Intensive Care, Amsterdam University Medical Centers, Location ‘AMC’, 1007 MB Amsterdam, The Netherlands

**Keywords:** difficult airway, physiological difficult airway, resource-limited setting, ICU, airway intervention

## Abstract

Airway management is central to the care of critically ill patients, yet it remains one of the most challenging interventions in emergency departments and intensive care units. Patients often present with severe physiological instability, limited cardiopulmonary reserve, and high acuity, while clinicians often work under constraints related to time for preparation, equipment availability, trained workforce, monitoring, and access to advanced rescue techniques. These challenges are particularly pronounced in low- and middle-income countries and other resource-limited or austere environments, where the margin for error is narrow and delays or repeated attempts in airway management may rapidly precipitate hypoxemia, hemodynamic collapse, or cardiac arrest. Although contemporary airway guidelines emphasize structured preparation and rescue pathways, many assume resources that are not consistently available in such settings. This narrative review discusses pragmatic, context-adapted strategies for airway management in constrained environments, with emphasis on physiology-first preparation, appropriate oxygenation and induction techniques, simplified rapid-sequence intubation, and the judicious use of basic airway adjuncts, supraglottic devices, and video laryngoscopy, where available. Adapted difficult airway algorithms, front-of-neck access in the absence of surgical backup, human factors, team training, and ethical considerations are also addressed. This review aims to support safer and effective airway management for critically ill patients in resource-limited emergency and intensive care settings.

## 1. Introduction

Airway management is a cornerstone of patient management in emergency and critical care. Yet it remains one of the most challenging and high-risk interventions, particularly in physiologically unstable patients. Although only a small proportion of emergency department (ED) patients, estimated at 0.5% to 1%, require intubation, these cases carry a disproportionately high risk of complications [1,2]. Difficult airway scenarios are not uncommon in emergency intubations, and the procedure itself is consistently associated with significant adverse events, including severe hypoxemia, hypotension, esophageal intubation, and cardiac arrest [2,3,4]. In critically ill patients, even brief interruptions in oxygenation or minor hemodynamic shifts can trigger rapid deterioration [5,6].

To mitigate these risks, airway management guidelines have been developed to promote safer practice [7,8,9]. The key principles in most guidelines include thorough airway assessment, physiological optimization, limiting the number of attempts, and structured as well as timely escalation to rescue strategies such as supraglottic airway placement (SGA) or front-of-neck access (FONA).

In well-resourced settings, such frameworks have contributed to more predictable and controlled intubation outcomes, particularly in operating theaters and intensive care units (ICUs) with specialized staff and access to required equipment. However, these protocols often assume an ideal infrastructure that includes resources such as video laryngoscopy, capnography, high-flow oxygen systems, various medication options, and multidisciplinary airway teams. However, in low- and lower-middle-income countries, as well as in high-income countries with peripheral and remote healthcare facilities, many of these resources are either unavailable or inconsistent [2,10]. Emergency airway management is frequently performed under less-than-ideal conditions, by non-specialists, with limited tools and minimal monitoring. These realities amplify the risks already inherent to critically ill patients and challenge the direct application of standard algorithms in such contexts.

For the purpose of this review, resource-limited (or low-resource) settings refer to clinical environments in which access to advanced airway equipment, continuous vital sign monitoring, experienced personnel, surgical backup, or post-intubation critical-care resources is inconsistent or unavailable. This includes emergency departments and ICUs in low- and lower-middle-income countries, as well as rural, remote, or austere settings where system constraints meaningfully influence decision-making.

This review explores how the core principles of difficult airway management, such as ensuring oxygenation, minimizing hemodynamic insult, and preparing for failure, can be adapted for resource-limited emergency and intensive care environments. We propose practical modifications to equipment, techniques, and planning, and introduce context-sensitive pathways that maintain the intent of established guidelines while addressing on-the-ground constraints. Our goal is to support clinicians in delivering safe and adequate airway care, even when working with limited resources.

## 2. Methods

### 2.1. Literature Search and Selection Criteria

This narrative review was conducted to develop a pragmatic framework for airway management in resource-limited critical-care settings. A structured literature search was performed in PubMed/MEDLINE, Embase, and the Cochrane Library. The search included English-language publications available up to 2025, incorporating recent international airway guidelines and consensus statements. Search terms included combinations of *difficult airway, critical care airway, Intensive Care Unit (ICU) intubation, physiologically difficult airway, cannot intubate cannot oxygenate (CICO), front–of–neck access (FONA), supraglottic airway, airway algorithms, airway guidelines, low–resource settings, and human factors*. Major international airway guidelines, consensus documents, and national airway audit reports were manually reviewed, and reference lists of key articles were screened to identify additional relevant publications.

### 2.2. Synthesis of Recommendations

Given the clinical focus and heterogeneity of available evidence, a narrative synthesis approach was used. Recommendations were evidence-informed and guidelines-aligned, when robust data were available. Where evidence was limited, particularly for workflows in resource-constrained ICU settings, recommendations were informed by expert consensus, clinical reasoning, and human-factors principles. The emphasis was placed on oxygenation-centered strategies, physiology-first decision-making, cognitive aids, and structured escalation, rather than device-specific mandates. Figures and algorithms explicitly indicate whether the content is derived from the published literature, guidelines, and/or expert consensus, and supporting references are provided in the figure legends.

### 2.3. Scope

This review does not propose a protocol but offers a context-adaptive framework intended to complement existing airway guidelines and support clinician decision-making in resource-limited emergency and critical-care settings.

## 3. Redefining the Scope of Airway Difficulty

**Figure 1:**
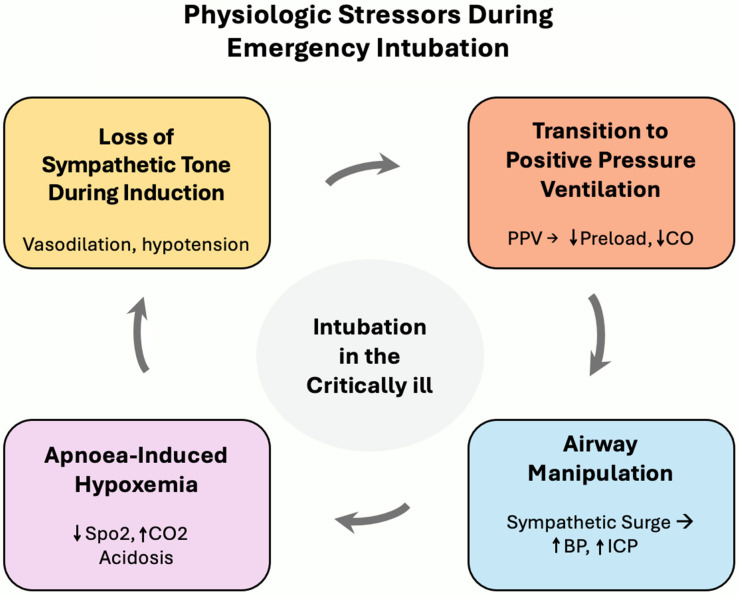
Physiologic stressors during emergency airway management in the critically ill. Emergency airway management exposes critically ill patients to sequential physiologic stressors, including loss of sympathetic tone during induction, apnea-related hypoxemia, hemodynamic effects of airway manipulation, and reduced venous return following initiation of positive-pressure ventilation. These stressors interact in a dynamic, self-reinforcing manner, whereby deterioration in one domain can amplify instability in others, creating a cascade of peri-procedural risk. The interaction of these factors contributes to peri-procedural instability in emergency settings. This figure is based on published physiologic principles and observational data describing peri-intubation instability in critically ill patients. CO: cardiac output, BP: blood pressure, ICP: intracranial pressure, SpO_2_: peripheral oxygen saturation, CO_2_: carbon dioxide.

The concept of a difficult airway has traditionally centered on anatomical challenges such as factors that impede glottic visualization, airway management, ventilation, and oxygenation [7,8]. In acute-care environments such as ED and ICUs, however, airway difficulty is increasingly recognized as a physiologic and contextual problem rather than a purely mechanical one (Figure 1). In these settings, airway management is challenging due to physiological perturbations which are mainly cardiorespiratory or metabolic in origin: limited reserves such as functional residual capacity causing rapid desaturation during apnea, induction drugs-related cardiovascular instability, and pathology-related spiral causing rapid deterioration. This may render even anatomically straightforward intubations and the initiation of positive-pressure ventilation hazardous.

The challenges of managing physiologically difficult airways are particularly consequential in resource-limited environments, where opportunities for optimization and resources for rescue are constrained (Table 1). Thus, airway management necessitates anticipating risk and planning that integrates physiologic vulnerability alongside anatomical assessment, supported by structured bedside risk stratification across anatomical, physiologic, and contextual domains (Table 2), with locally available resources [11,12,13,14].

## 4. Core Principles of Difficult Airway Management

Despite differences in geography and institutional context, contemporary difficult airway guidelines share core principles centered on structured preparation, optimizing oxygenation and ventilation, first-pass success, and a staged response to airway management or ventilation failure [7,8,9]. These practices aim not only to facilitate successful airway control but also to mitigate hypoxia, hemodynamic collapse, and the need for invasive procedures.

### 4.1. Pre-Intubation Assessment and Planning

**Figure 2:**
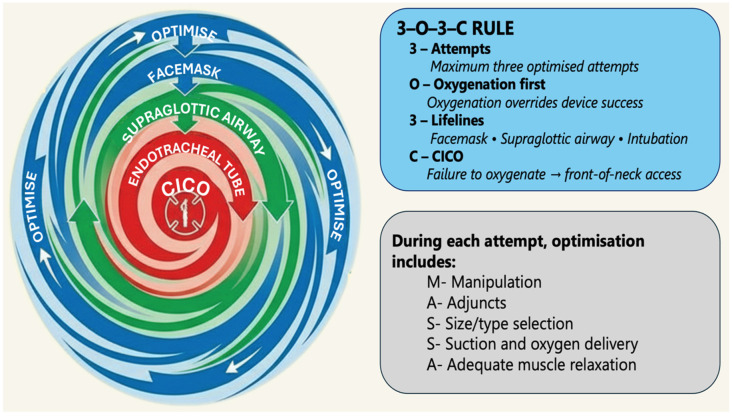
The Vortex approach to emergency airway management. The Vortex approach is a cognitive aid designed to prioritize oxygenation and mitigate cognitive overload during emergency airway management. It conceptualizes airway management as progression through three oxygenation lifelines—facemask ventilation, supraglottic airway insertion, and tracheal intubation—with optimization at each attempt. Failure to maintain oxygenation mandates early transition to front-of-neck access. This figure is adapted from the published literature describing the Vortex cognitive aid and is supported by international airway guidelines. CICO: cannot intubate, cannot oxygenate.

Pre-intubation risk evaluation is fundamental to safe airway management. Recent guidelines emphasize identifying risk factors for difficult laryngoscopy, bag-mask ventilation, the need for early SGA use, and FONA. Structured tools such as the LEMON mnemonic (look externally, evaluate 3–3–2 rule, Mallampati score, obstruction, neck mobility), MACOCHA score (used predominantly in ICU settings), and the upper lip bite test (ULBT), are commonly employed, though their validation and formal incorporation into guidelines vary [8,15,16]. These tools support rapid bedside assessments but should be interpreted within a clinical context. In parallel, contemporary airway management frameworks such as the Vortex approach (Figure 2) extend beyond anatomical assessment to explicitly incorporate situational and human factors, including urgency, environmental constraints, team composition, resource availability, and airway planning [17]. Collectively, these approaches emphasize the role of structured pre-intubation assessment in anticipatory airway planning.

### 4.2. Oxygenation and First-Pass Success

Maintaining adequate oxygenation during airway management is a universally recognized and prioritized imperative in both elective and emergency settings. Preoxygenation utilizing non-invasive ventilation (NIV) or high-flow nasal oxygen (HFNO) effectively extends the duration of the safe apnea window [18]. Techniques such as apneic oxygenation (leaving low-flow nasal cannula or HFNO in place, during laryngoscopy) are simple yet effective adjuncts [19]. Video laryngoscopy is increasingly recommended as a first-line tool, supported by evidence that it improves glottic view and reduces failed attempts. The guidelines strongly advocate for achieving first-pass success by utilizing the most experienced operator and employing the most effective technique during the initial attempt.

### 4.3. Algorithmic Structure and Decisive Progression

**Figure 3:**
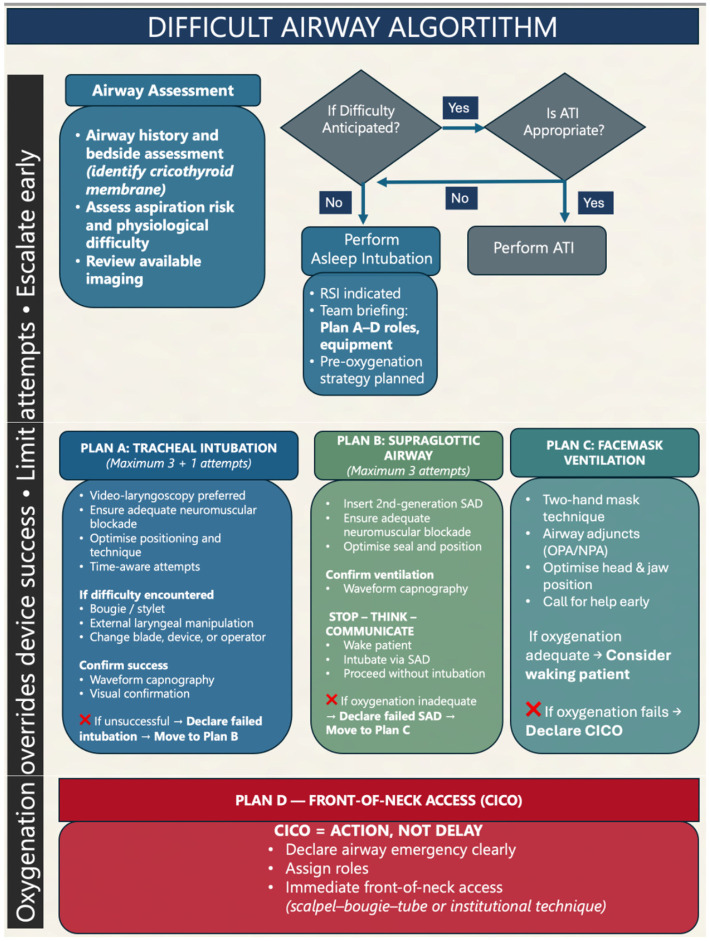
Difficult airway algorithm. A stepwise, oxygenation-centered pathway incorporating early risk assessment, attempt limitation, and timely escalation to rescue strategies, including front-of-neck access in CICO situations. ATI: awake tracheal intubation; RSI: rapid-sequence intubation; SGA: supraglottic airway; SAD: supraglottic airway device; OPA: oropharyngeal airway; NPA: nasopharyngeal airway; CICO: cannot intubate, cannot oxygenate. This algorithm is derived from established international airway guidelines and consensus recommendations, adapted for emergency and critical-care settings.

All major airway algorithms adopt a structured, tiered approach commonly referred to as Plans A through D to minimize delays and reduce cognitive overload (Figure 3). Plan A represents the initial intubation attempt using the most experienced provider and the best available technique (often video laryngoscopy). If this fails, Plan B is to insert a SGA to re-establish oxygenation. Plan C serves as a fallback: attempting facemask ventilation or if feasible, awakening the patient. If ventilation remains inadequate, Plan D calls for emergency FONA, most commonly via a scalpel–bougie cricothyrotomy, a technique endorsed by the current Difficult Airway Society (DAS) guidelines for its speed and effectiveness. Prompt progression through each step is essential; clinicians are strongly advised not to persist with repeated, unsuccessful attempts that risk worsening hypoxia and airway trauma [7,8].

### 4.4. Confirmation and Monitoring

Continuous waveform capnography is the gold standard for confirming endotracheal tube placement and monitoring ventilation. Its absence has been a key factor in adverse outcomes, as highlighted in the Seventh National Audit Project (NAP7) of the Royal College of Anaesthetists, which examined major airway and respiratory complications associated with airway management [20].

### 4.5. Human Factors and Team Coordination

Effective airway management is as much about teamwork as it is about technique. Guidelines emphasize early activation of skilled help, clear role assignments, and the use of cognitive aids or checklists to guide actions under pressure and to reduce cognitive load [21]. The DAS 2025 update, in particular, reinforces the importance of simulation training, team briefings, and attention to human factors as key components of crisis resource management [8].

### 4.6. Awake Intubation and Special Considerations

When anatomical or physiologic difficulty is anticipated, such as an inability to tolerate apnea or predicted mask ventilation failure, awake intubation is often the safer approach. Recent guidelines present a decision tree to assist in identifying such scenarios, advocating awake intubation when spontaneous ventilation must be preserved or when surgical airway access may be especially difficult [7,8]. Awake fiberoptic or video laryngoscopy allows troubleshooting in real-time while maintaining oxygenation and ventilation [22].

## 5. Barriers to Guideline Implementation in Low-Resource Settings

**Figure 4:**
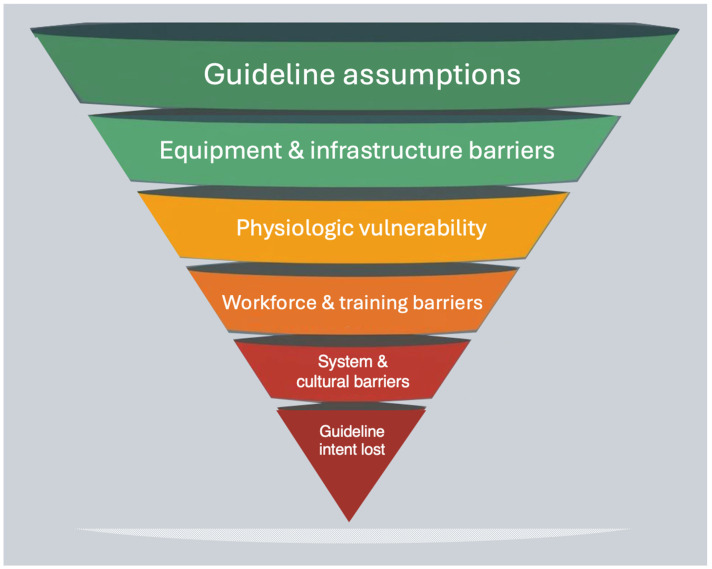
Pyramid of barriers to difficult airway guideline implementation in low-resource ED and ICU settings. The pyramid illustrates cumulative barriers to airway guideline implementation in low-resource environments, ranging from equipment and infrastructure limitations to workforce, system, and cultural constraints. As these layers converge, the assumptions underlying standard airway algorithms fail, leading to loss of guideline intent during real-world emergencies. This conceptual model represents a narrative synthesis of the published literature and health-system surveys describing barriers to airway management in resource-limited emergency and critical-care settings.

There are considerable challenges to the availability of airway equipment and guidance on difficult airway management in low-resource EDs and ICUs (Figure 4) [2,23,24].

### 5.1. Equipment and Infrastructure Constraints

A fundamental barrier is the mismatch between guideline assumptions and on-the-ground equipment availability. Most contemporary airway algorithms assume unlimited access to continuous waveform capnography, video laryngoscopy, multiple sizes of SGA, and readily available FONA equipment [7,8]. In many low- and lower-middle-income settings, these resources are unavailable, intermittently functional, or restricted to selected locations.

Surveys from sub-Saharan Africa and South Asia consistently demonstrate limited access to core airway-safety tools. In a national Ugandan survey of surgical and critical-care facilities, waveform capnography was present in fewer than 20% of operating rooms, video laryngoscopes in less than 5%, and dedicated cricothyrotomy kits were in only a small minority of hospitals [25]. Similar gaps have been reported across South Asian EDs and ICUs, where recommended airway monitoring and rescue devices, such as waveform capnography, video laryngoscopes, and second-line airway adjuncts, are inconsistently available or used, particularly during emergency and out-of-theater intubations [24,26,27,28,29].

Oxygen infrastructure represents another area of concern. While international guidelines emphasize continuous oxygenation using HFNO, non-invasive ventilation, and apneic oxygenation reliable oxygen delivery remains inconsistent in many low-resource institutions [30,31]. Limitations in physiologic monitoring further compound the risk, delaying recognition of deterioration during airway management.

### 5.2. Workforce and Training Limitations

Workforce realities amplify equipment constraints. Many low-resource health systems operate with a severe shortage of trained clinicians and competent airway operators such as anesthesiologists and intensivists [24]. Thus, airway management in emergency and ICU settings is frequently performed by junior physicians, generalists, or non-physician providers [32]. Task-shifting is often unavoidable and often introduces substantial variability in airway expertise, particularly for advanced rescue techniques [33].

This mismatch becomes the most apparent during rare but potentially catastrophic events, such as cannot intubate, cannot oxygenate (CICO) scenarios. Providers practicing in environments without routine access to cricothyrotomy kits or structured airway training frequently report minimal simulation-based or hands-on experience with surgical airway techniques [34]. As a result, those more likely to encounter airway failure are often less prepared to execute definitive rescue.

### 5.3. System and Cultural Barriers

Beyond tangible resource constraints, system-level and cultural factors further limit guideline adherence [35,36]. Formal airway risk stratification, pre-intubation checklists, and structured team briefings are seldom embedded into routine emergency workflows, particularly in high-volume, time-critical environments. As a result, airway planning is often implicit rather than explicitly shared, increasing the risk of omission and fixation errors.

Difficult airway encounters are frequently under-documented, and handover of airway risk between shifts or during inter-facility transfer is inconsistent [36]. Moreover, nationwide databases or registries for difficult airways are mainly limited to high-income countries or academic institutions. Thus, communication failures remain a common concern with no reliable dissemination of information among healthcare professionals regarding airway management [37]. The hierarchical team dynamics further influence airway behavior, with junior team members often hesitant to express concern, prompt escalation, or declare failure when senior clinicians persist with repeated laryngoscopy attempts, contrary to guideline emphasis on early failure recognition and timely rescue [38].

Taken together, these constraints explain why direct transposition of high-income-country airway algorithms is often impractical in low-resource ED and ICU settings. The limitation lies not in the quality of existing guidelines, but in their misalignment with local realities.

## 6. A Pragmatic Approach to Difficult Airway Management

Having outlined the barriers that limit guideline implementation, the following sections focus on how core airway-safety principles can be operationalized despite these constraints.

### 6.1. Rapid Bedside Risk Stratification

In low-resource EDs and ICUs, early identification of a high-risk airway is a decisive safety step. In the context of limited monitoring, constrained rescue capacity, and physiological instability, airway assessment must prioritize speed, clarity, and actionability over detailed scoring or formal prediction.

When risk indicators are present across multiple domains (Table 3), the airway management should be considered high-risk, regardless of the anticipated laryngoscopy grade [39]. In such situations, a rapid bedside framework that integrates these domains may help consolidate clinical judgment. The HEAVEN criteria (hypoxemia, extremes of size, anatomic challenge, vomit/blood, exsanguination, neck mobility) provide such a framework by combining anatomical and physiological risk factors to support early identification of high-risk airways in emergency settings [40]. This integration is particularly relevant in resource-constraint EDs and ICUs, where contextual limitations further narrow the margin for error and physiological instability frequently precedes technical failure. Rather than serving as a tool to predict intubation difficulty, HEAVEN supports early risk designation, reinforcing the need for first-attempt optimization, strict limitation of repeated attempts, and advanced preparation for rescue strategies. Used in conjunction with a domain-based assessment, such frameworks help ensure that high-risk airways are recognized *before induction* rather than identified only after airway failure.

### 6.2. Pre-Oxygenation and Peri-Intubation Oxygenation Without Advanced Devices

Conventional rapid-sequence induction paradigms, largely extrapolated from operating room practice, assume preservation of known cardiorespiratory reserve, adequate invasive monitoring, and immediate access to vasoactive support. These assumptions are often invalid in resource-constrained environments. Induction in the critically ill must, therefore, be approached as a high risk physiologic transition, rather than a purely technical prelude to laryngoscopy. The objective should be pragmatic, minimizing hypoxia, hypotension, and apnea time, and maximizing the likelihood of first-pass success.

The maintenance of oxygenation remains the overriding safety objective during emergency airway management, particularly once a high-risk airway has been identified. Although advanced oxygenation modalities such as HFNO or NIV interfaces are often unavailable, their absence does not preclude adequate oxygenation when fundamental principles are applied deliberately.

In practice, failure of an attempt to restore oxygen delivery should, therefore, be regarded as a safety-preserving action rather than a procedural failure. In low-resource environments, peri-intubation hypoxemia most commonly results from inadequate mask seal, insufficient oxygen delivery, suboptimal positioning, or prolonged apnea—factors that remain modifiable even in the absence of advanced equipment. A limited number of low-cost, high-yield interventions can substantially improve the oxygen reserve and reduce the desaturation risk [31,41,42]. These measures focus on optimizing basic ventilation mechanics, maximizing inspired oxygen concentration, and minimizing unventilated time, and are summarized in Table 4.

Strict adherence to a no-ventilation rapid-sequence induction paradigm is frequently unsafe in critically ill patients with limited physiological reserve. Evidence from ED and ICU cohorts indicates that gentle, pressure-limited bag-mask ventilation between attempts reduces severe hypoxemia without a corresponding increase in clinically significant aspiration, particularly in patients with shock or severe respiratory failure.

As an adjunct to these core strategies, apneic oxygenation using a nasal cannula may be employed during laryngoscopy. Even in low-resource settings, a standard nasal cannula set at 10–15 L/min after preoxygenation can provide continuous oxygen delivery during apnea. Randomized trials in critically ill adults have demonstrated inconsistent effects on the lowest oxygen saturation during apnea, but lack an identified harm [43]. Accordingly, apneic oxygenation should be viewed as a low-risk safety adjunct rather than a substitute for effective preoxygenation or ventilation, and is commonly maintained during intubation attempts as an additional margin of safety.

### 6.3. Induction Strategies in Hemodynamically Fragile Patients

Agents with relative cardiovascular stability, most notably ketamine and etomidate, are preferred, while propofol should be avoided or used only in markedly reduced doses when alternatives are unavailable [44]. However, the dose titrated to the effect, rather than the choice of agent, is more important for a critically unwell patient [5] (Table 5).

A reluctance to use neuromuscular blockade is common in low-resource settings because of a lack of experienced airway operators and limited availability of antidotes such as sugammadex. However, inadequate sedation without paralysis leads to prolonged attempts, increased oxygen consumption, and airway trauma. When intubation is required, decisive paralysis improves first-pass success and reduces cumulative physiologic insult, provided rescue strategies are prepared in advance. The absence of immediate pharmacologic reversibility should not outweigh the risk of an uncontrolled airway [45,46].

The administration of a vasopressor via infusion or push-dose should be routine, as even brief hypotension may precipitate cardiovascular collapse. The use of a fluid bolus versus a vasopressor to mitigate preinduction hypotension should be individualized based on the patient’s condition. Induction strategies in low-resource settings must, therefore, be physiology-driven, include large-bore IV access with adequate monitoring such as frequent cycling of non-invasive blood pressure, and be explicitly adapted to constrained rescue capacity.

### 6.4. Device Strategy When Equipment Is Limited

**Figure 5:**
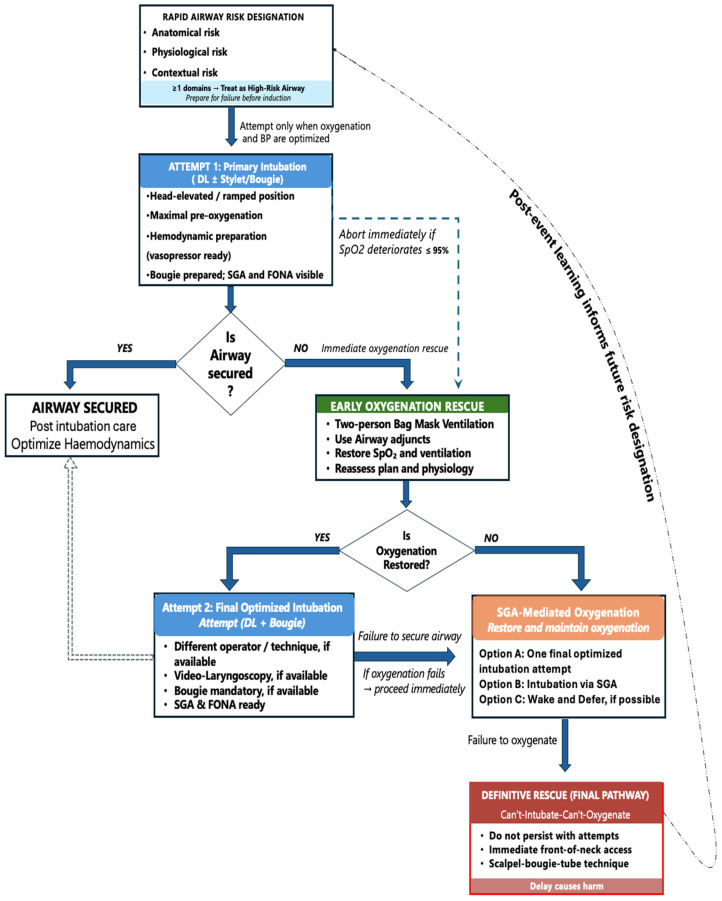
Context-adapted difficult airway algorithm for low-resource emergency department and intensive care unit settings. The algorithm prioritizes early bedside risk designation, explicit limitation of intubation attempts, and escalation driven by physiological deterioration—particularly hypoxemia—rather than repeated device failure. Branching complexity is minimized to reduce cognitive load under stress, with supraglottic airway placement positioned early as a reliable oxygenation rescue strategy and front-of-neck access framed as a definitive, time-critical intervention. Advanced devices (e.g., video laryngoscopy, bronchoscopy) function as optional modifiers when locally available but are not required for safe execution of the pathway. This figure presents a context-adapted airway management algorithm derived from established airway guidelines and informed by published observational evidence and audit data from emergency and critical-care settings.

A lack of availability of video laryngoscopes mandates the use of direct laryngoscopy as the primary intubation technique in many low-resource settings [24]. It is, thus, imperative to optimize the conditions, including appropriate positioning, external laryngeal manipulation, and the use of adjuncts such as a bougie or an endotracheal tube with a stylet, to increase first-pass success [47,48]. Observational data from ED and ICU cohorts consistently demonstrate that repeated laryngoscopy attempts are associated with progressively increasing rates of hypoxia, hypotension, and cardiac arrest. Although some guidelines permit up to three intubation attempts, complication rates rise sharply beyond the second attempt [49]. In low-resource settings, an early call for help and adopting of limited attempts (≤2-attempt threshold) represents a safety-oriented operationalization of guideline intent rather than a departure from it (Figure 5).

SGA devices represent the most reliable immediate rescue for oxygenation failure in this context [7,8,9]. Although second-generation SGAs are recommended in contemporary guidelines because of improved seal and gastric access, their availability in low-resource settings is often limited to first-generation devices, which often have a restricted size range and minimal device redundancy [24,25,26,27,28]. Despite these constraints, early insertion of an available SGA following failed laryngoscopy reliably restores ventilation, mitigates hypoxemia, and creates time for reassessment. The maintenance of adequate oxygenation should, therefore, be regarded as a successful interim priority, regardless of whether a definitive airway is immediately achieved [46].

Where video laryngoscopy is available as a shared or limited resource, selective deployment for predicted anatomically difficult or physiologically high-risk airways is preferable, as it reduces cognitive load, and improves first-pass success. When oxygenation cannot be restored, FONA remains the definitive life-saving intervention. The absence of commercial kits should not delay action; an open surgical cricothyrotomy using basic instruments such as a scalpel, bougie or finger, and tube, remains the most reliable technique. Across major airway audits and ICU cohorts, delayed progression to FONA remains a leading contributor to airway-related mortality, most commonly due to hesitation rather than a lack of equipment [49,50] (Table 6).

### 6.5. Implementing Safe Airway Practice in Resource-Constrained Emergency and Critical Care

**Figure 6:**
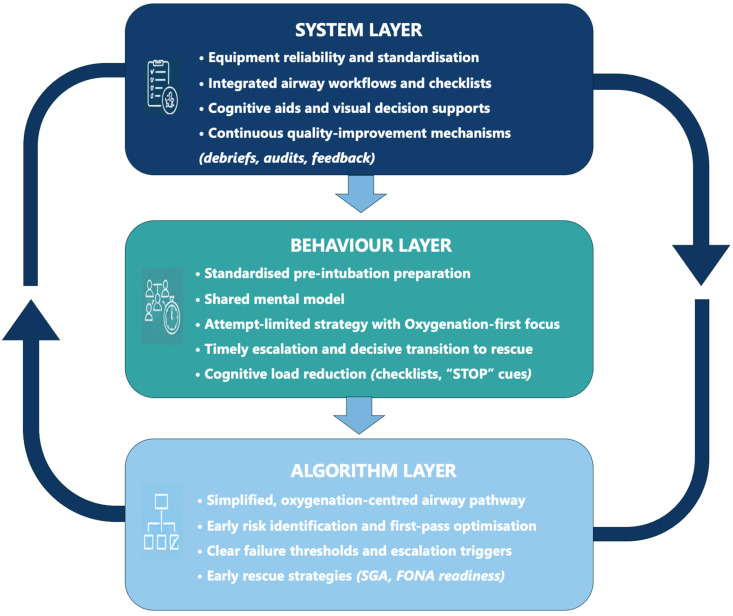
Building a resilient airway management system through integrated pillars. The figure presents a three-layer framework for airway safety in emergency and critical-care settings. The *system layer* provides structural support through equipment reliability, workflow integration, cognitive aids, and continuous quality-improvement mechanisms. The *behavior layer* represents reproducible team practices that govern human performance during airway management, including standardized preparation, shared mental modeling, attempt limitation, and timely escalation to rescue strategies. The *algorithm layer* structures cognitive decision-making by defining failure thresholds and oxygenation-centered transitions to rescue. Alignment across these pillars enhances resilience and reduces airway-related adverse events, particularly in resource-constrained environments. This framework is based on the human-factors literature and expert consensus, integrating system, behavioral, and algorithmic components of airway safety.

A context-adapted difficult airway algorithm improves outcomes only when it is reliably enacted at the bedside. In low-resource environments, the principal threats to airway safety arise less from deficiencies in algorithms and more from inconsistent execution, variable team readiness, equipment unreliability, and delayed escalation. Translating a simplified, physiology-anchored algorithm into practice, therefore, requires operational systems that reinforce predictable behaviors [48]. The central objective is to ensure safety prioritization, maintaining oxygenation, recognizing failure early, and progressing rapidly to an available resource-based shared plan, regardless of operator experience or equipment availability. The interaction between the algorithm design, clinician behavior, and system-level reliability is summarized in the three-layer implementation framework shown in Figure 6.

### 6.6. Prioritizing Executability over Completeness

Effective implementation begins not with an inventory of ideal resources but with an assessment of what can be reliably performed, every time (Table 7). Systems designed around executability consistently outperform more elaborate pathways in settings with high staff turnover and heterogeneous skill levels. A minimal, always-available airway It kit, comprising a functioning laryngoscope, an endotracheal tube, a bougie, a single reliable supraglottic airway, working suction, a dependable oxygen source, and a scalpel–bougie–6.0 endotracheal tube set for emergency FONA forms the foundation of this approach [23]. Alongside equipment readiness, three actions must be standardized before the first attempt: achieving physiologic positioning, maximizing oxygenation, and ensuring hemodynamic preparedness, including vasopressor availability. Embedding a predefined attempt rule into routine workflow is equally important; large audits such as the Fourth National Audit Project (NAP4) of the Royal College of Anaesthetists and ICU airway registries demonstrate that complication rates rise sharply beyond two attempts. At the same time the likelihood of successful intubation falls [23,47,49]. Making this “predefined attempt limit” explicit in team communication transforms escalation from a subjective judgment into a predictable safety behavior. [23,49,51].

### 6.7. Training Focused on Recognition, Escalation, and Oxygenation Strategy

Traditional airway training focuses heavily on devices and technique. In contrast, low-resource systems benefit most from training that emphasizes decision-making, specifically, when to pause and think to reduce cognitive load and when to abandon further attempts. Frequent training of stakeholders includes using micro-simulations that rehearse early declaration of a failed attempt, timely supraglottic rescue, and execution of a scalpel–bougie cricothyrotomy on basic models. Short oxygenation drills, including teaching on effective mask seal, rapid reoxygenation between attempts, and immediate transition from mask to SGA, improve practical performance more than sporadic high-fidelity simulation [46]. Evidence from simulation science and the emergency airway literature supports this “low-dose, high-frequency” approach, showing improved retention of critical behaviors and significant reductions in severe peri-intubation hypoxemia [52]. In practice, these drills shift training away from mastering multiple devices and toward mastering the cognitive transitions that prevent physiologic collapse.

### 6.8. Cognitive Aids, Checklists, and Team Behaviors

Human-factors research consistently demonstrates that simple, well-designed cognitive aids reduce omission errors, enhance shared situational awareness, and accelerate the transition to effective rescue during airway crises. In low-resource environments, these tools provide high-value, low-cost safety supports by stabilizing preparation and reducing cognitive load [53]. Brief, structured aids, such as pre-intubation checklists, airway briefing cards, and oxygenation-failure prompts, reinforce key steps in first-pass optimization and escalation. Their impact on reducing physiologic complications, particularly hypoxemia, is well documented across ICU airway audits. Training the team on a locally adapted checklist enhances shared decision-making and reduces the likelihood of communication breakdown during critical events.

Equally important are the behavioral components that translate the algorithm into predictable bedside execution [54]. A short shared mental model script stating Plans A–D aloud aligns all team members around the intended pathway, while clear role assignment reduces fragmentation during escalation. Empowering any team member to initiate a “stop–oxygenate” call mitigates hierarchical delay, and integrating a 20-second laryngoscopy time-check reduces task fixation. Together, these cognitive and behavioral supports strengthen adherence to physiologic priorities and enhance the reliability of airway management under low-resource conditions. Detailed examples of high-value cognitive aids are provided in Table 8.

### 6.9. Equipment Reliability and Workflow Integration

In many low-resource systems, equipment failure, along with the absence of equipment, is the more significant hazard. Ensuring reliability requires embedding readiness-checks into the routine workflows rather than relying on ad hoc inspection. Daily verification of the airway cart, color-coded organization of equipment trays, and standardized, sealed “grab and go” airway kits reduce cognitive load and prevent catastrophic delays during emergencies. These interventions parallel improvements observed in trauma systems and resuscitation cart redesign studies, where structured organization consistently improves the response time and adherence to clinical pathways. A consolidated overview of practical system-level interventions that support safe airway management in resource-limited settings is presented in Table 9.

### 6.10. Learning from Events and Sustaining Change

Long-term improvement depends on structured learning cycles. Regular, non-punitive review of difficult airway events such as focusing on recognition of hypoxia, timing of escalation, and adherence to the algorithm, creates a feedback loop that strengthens system performance. Monitoring key metrics, such as the number of attempts, hypoxemia episodes, FONA timing, and unplanned ICU transfers, provides actionable data to refine local practice. Annual revision of the context-adapted algorithm ensures that it evolves alongside real-world experience. This continuous quality improvement model mirrors the approach taken by national audits such as NAP4, where iterative learning has been central to advancing airway safety.

## 7. Ethical and Legal Considerations in Resource-Limited Airway Management

Emergency airway management in resource-limited settings presents unique ethical challenges, particularly when clinicians must balance individual patient benefit against system constraints and competing clinical priorities. Decisions regarding airway escalation, the use of invasive rescue techniques, and the allocation of limited personnel or equipment often occur under time pressure and uncertainty.

Ethical considerations include proportionality of intervention, avoidance of preventable harm, and respect for patient dignity, especially in situations where the likelihood of successful rescue is low or downstream critical-care resources are unavailable. In such contexts, clear pre-procedural planning, team communication, and documentation of decision-making rationale are essential.

This review emphasizes that context-adapted airway strategies should be applied within existing legal and institutional frameworks, with escalation decisions guided by patient physiology, anticipated benefit, and available resources rather than technical capability alone. Incorporating ethical awareness into airway planning supports transparent, defensible, and patient-centered decision-making in high-risk emergencies.

## 8. Limitations

There are several limitations of this review. First, the available literature on difficult airway management in critically ill patients is heterogeneous, comprising guidelines, audits, observational studies, and expert consensus, with limited high-quality data specific to resource-limited critical-care settings. Consequently, some recommendations rely on contextual adaptation and expert judgment. In addition, regional variability in resources, training, and healthcare systems may influence the applicability of the proposed framework, which is intended to complement, rather than replace, local protocols and clinician decision-making. Finally, this review is narrative in nature and we did not conduct a systematic review of the literature on airway management in resource-limited settings; and a few recommendations may lack evidence or generalizability.

## 9. Refined Future Directions

Future research should prioritize context-aware airway-safety strategies in resource-limited emergency and critical-care settings. Key areas include validating physiology-based risk stratification tools, evaluating of low-cost oxygenation and monitoring solutions, and assessing simplified airway system bundles that integrate equipment readiness, cognitive aids, and team behaviors. In addition, implementation science approaches are needed to examine how guideline principles can be reliably translated into practice across diverse healthcare environments. Strengthening the evidence base in these domains will support scalable, sustainable improvements in airway safety beyond high-resource settings.

## 10. Conclusions

Difficult airway management in emergency and critical-care settings is fundamentally a problem of safety under uncertainty. In low-resource environments, this uncertainty is magnified by constrained equipment, variable expertise, fragile systems, and patients with limited physiologic reserve. Direct transposition of airway algorithms developed in high-income countries into such contexts often fails, not because the principles are flawed, but because their execution presumes conditions that are not reliably present.

This review demonstrates that adequate airway safety in low-resource ED and ICU settings depends on preserving the core intent of established guidelines while simplifying their application. By anchoring decision-making to oxygenation, emphasizing early recognition of failure, limiting attempts, and prioritizing reliable rescue strategies, airway management can be rendered both safer and more reproducible despite constrained resources. Context-adapted risk stratification, physiology-driven induction strategies, pragmatic device sequencing, and streamlined, oxygenation-focused algorithms collectively reduce cognitive load and delay in escalation. Pragmatic adaptation, embedded within local training and systems, offers a viable pathway to closing the gap between guideline intent and bedside practice.

## Figures and Tables

**Table 1:** Physiologically difficult airway scenarios and key management principles.

Clinical Scenario	Primary Physiologic Risk	Key Management Strategies
Hypovolemic shock	Loss of preload Circulatory collapse with induction or PPV	Fluid or blood resuscitation Vasopressors ready before induction Reduced or target-based induction dosing (e.g., ketamine)
Severe metabolic acidosis (e.g., DKA)	Rapid desaturation during apnea CO_2_ retention and worsening acidosis	Optimize acid–base status with resuscitation Minimize apnea time Consider apneic oxygenation
Right ventricular failure (e.g., PE)	Sensitivity to preload and afterload changes Increased PVR Reduced CO	Vasopressors (e.g., epinephrine, vasopressin) Avoid high tidal volumes and excessive PEEP Consider inhaled pulmonary vasodilators
Neurocritical patient	Raised intracranial pressure from laryngoscopy Reduced cerebral perfusion pressure	Opioid premedication (e.g., fentanyl) Maintain MAP Target-based induction dosing Avoid ICP surges
Profound hypoxemia	Rapid desaturation High risk of peri-intubation arrest	Aggressive preoxygenation NIV or HFNO where available Apneic oxygenation Early, controlled intubation
Cardiogenic shock	Limited cardiac reserve Hemodynamic instability with sedatives and PPV	Vasopressors/inotropes prepared Reduced induction dose Avoid excessive PEEP Close hemodynamic monitoring
Septic shock	Induction-related hypotension Vasoplegia Relative adrenal insufficiency	Early vasopressors Judicious fluid resuscitation Stress-dose steroids where indicated

CO: cardiac output; CO_2_: carbon dioxide; DKA: diabetic ketoacidosis; HFNO: high-flow nasal oxygen; ICP: intracranial pressure; MAP: mean arterial pressure; NIPPV: non-invasive positive-pressure ventilation; PE: pulmonary embolism; PEEP: positive end-expiratory pressure; PPV: positive-pressure ventilation; PVR: pulmonary vascular resistance.

**Table 2:** Expanded classification and identification of difficult airway types.

Type of Difficulty	Common Causes/Features	Bedside Identification Tools
Anticipated difficult intubation	Restricted mouth opening Reduced neck mobility Facial or airway trauma Obesity	MACOCHA score Mallampati classification Upper lip bite test (ULBT) Inter-incisor distance Thyromental distance
Difficult bag-mask ventilation	Obesity Facial hair Edentulism Upper airway obstruction Reduced lung compliance	MOANS mnemonic
Difficult laryngoscopy	Limited neck extension Large tongue Facial or upper airway trauma	Mallampati score ULBT LEMON assessment
Difficult airway management	Poor glottic view Blood or secretions Distorted anatomy Multiple failed attempts	Cormack–Lehane grade Percentage of glottic opening (POGO) Repeated failed laryngoscopy
Difficult supraglottic airway use	Limited mouth opening Upper airway masses or obstruction	Resistance to insertion Inadequate ventilation after placement
Difficult front-of-neck access	Obesity Distorted neck anatomy Previous neck surgery or trauma	Poor landmark palpation Difficult ultrasound identification
Physiologically difficult airway	Shock Severe hypoxemia Metabolic acidosis RV failure Raised ICP	Vital signs (MAP, SpO_2_) ABG (pH, PaCO_2_) Lactate Focused cardiac ultrasound Pre-intubation Shock Index

ABG: arterial blood gas; ICP: intracranial pressure; LEMON: look externally, evaluate 3–3–2 rule, Mallampati score, obstruction, neck mobility; MACOCHA: Mallampati score, apnea syndrome, cervical spine limitation, opening of mouth < 3 cm, coma, hypoxemia, anesthesiologist non-availability; MAP: mean arterial pressure; MOANS: mask seal, obesity, age > 55, no teeth, stiff lungs; PaCO_2_: partial pressure of arterial carbon dioxide; POGO: percentage of glottic opening; RV: right ventricle; SpO_2_: peripheral oxygen saturation; ULBT: upper lip bite test.

**Table 3:** Airway and physiologic risk screen for low-resource emergency department and intensive care unit settings.

Risk Domain	High-Risk Indicators (Any Present)	Clinical Implication
Anatomical	Facial or neck trauma Airway burns or edema Limited mouth opening Restricted neck movement Obesity with large neck circumference Stridor or upper airway obstruction	Increased risk of difficult mask ventilation and/or laryngoscopy
Physiological	SpO_2_ < 90% despite oxygen Severe pneumonia or ARDS Shock or hypotension Severe metabolic acidosis Sepsis Profound anemia	Reduced tolerance to apnea and induction-related cardiopulmonary instability
Contextual/System	No video laryngoscope Absence of capnography Limited SGA availability or sizes No ready FONA setup Limited senior airway support	Reduced margin for rescue and delayed recovery after airway failure

Note: Classification as a physiologically high-risk airway is warranted when clinically significant risk is present in two or more domains. In such cases, management should prioritize first-attempt optimization, strict limitation of intubation attempts, and upfront preparation for SGA rescue and FONA before induction, with early escalation as required. ARDS—acute respiratory distress syndrome; ED—emergency department; FONA—front-of-neck access; ICU—intensive care unit; SGA—supraglottic airway; SpO_2_—peripheral oxygen saturation.

**Table 4:** Pragmatic pre-oxygenation and peri-intubation oxygenation strategies for low-resource ED/ICU.

Strategy	Practical Application with Basic Resources	Primary Benefit
Two-person mask technique	Two-hand bag-mask ventilation	Improves mask seal and delivered FiO_2_
Reservoir-based oxygen delivery	BVM or non-rebreather mask with reservoir at maximal flow	Increases alveolar oxygen stores
Apneic oxygenation	Standard nasal cannula (10–15 L/min) left in place during laryngoscopy	Delays desaturation during apnea
Head-elevated/ramped position	Upper torso elevated ~20–30°	Improves FRC and laryngoscopy conditions
PEEP via BVM (if available)	Simple PEEP valve (5–10 cm H_2_O) on BVM	Reduces alveolar collapse
Brief NIV pre-oxygenation	Short CPAP/BiPAP in cooperative severe hypoxemia	Recruits lung units
Early re-oxygenation	Early pause in laryngoscopy to resume mask ventilation	Prevents critical desaturation

BiPAP: bilevel positive airway pressure; BVM: bag–valve–mask; CPAP: continuous positive airway pressure; FiO_2_: fraction of inspired oxygen; FRC: functional residual capacity; NIV: non-invasive ventilation; PEEP: positive end-expiratory pressure.

**Table 5:** Context-adapted induction strategies for hemodynamically fragile patients.

Aspect	Recommended Approach	Rationale in Low-Resource Settings
Induction agent	Ketamine (1–2 mg/kg IV) or Etomidate (0.2–0.3 mg/kg IV)	Relative cardiovascular stability in shock and hypoxemia
Agents to avoid/limit	Propofol (avoid or use reduced dose)	Vasodilation and myocardial depression
Neuromuscular blockade	Full-dose succinylcholine or rocuronium	Improves first-pass success; reduces airway trauma
Concern about reversibility	Secondary to airway control	Failed airway more dangerous than prolonged paralysis
RSI technique	Modified RSI with gentle mask ventilation if hypoxemic	Reduces severe desaturation
Pre-induction optimization	Volume assessment; vasopressor ready	Prevents peri-intubation hypotension
Monitoring	Frequent NIBP/manual BP checks if invasive unavailable	Early detection of hemodynamic collapse
Abort criteria	Early pause to reoxygenate or support BP	Oxygenation failure triggers escalation

BP: blood pressure; NIBP: non-invasive blood pressure; RSI: rapid-sequence induction.

**Table 6:** Context-adapted device strategy for low-resource environment.

Airway Step	Preferred Strategy	Rationale
Primary intubation	Optimized direct laryngoscopy	Universally available; effective when optimized
Adjunct use	Early bougie or stylet	Improves first-pass success
Attempt limitation	≤2 optimized attempts	Reduces trauma and hypoxemia
Rescue oxygenation	Early supraglottic airway	Rapid restoration of oxygenation
Definitive rescue	Surgical cricothyrotomy	Only definitive solution in CICO
Video laryngoscope	Selective use in high-risk airways	Preserves availability and expertise
Fiberoptic scope	Not central to core pathway	Avoids dependence on scarce devices

CICO: cannot intubate, cannot oxygenate.

**Table 7:** Minimal high-value airway equipment set for low-resource ED/ICU.

Essential (“Must-Have”)	High-Value (Optional/When Available)
Laryngoscope with spare blades/batteries	Video laryngoscope (shared)
Endotracheal tubes (6.0–8.0 mm)	Additional SGA sizes
Bougie or introducer	Second-generation SGA
Bag–valve–mask with reservoir	HFNO or NIV interface
Supraglottic airway (sizes 3–4)	Commercial cricothyrotomy kit
Suction device	Capnography
Basic FONA tray (scalpel, ETT)	Flexible bronchoscope

ETT: endotracheal tube; FONA: front-of-neck access; HFNO: high-flow nasal oxygen; NIV: non-invasive ventilation; SGA: supraglottic airway.

**Table 8:** High-value cognitive aids for low-resource ED/ICU airway management.

Cognitive Aid/Tool	Purpose and Key Elements	Evidence Base/Rationale
PEARL Pre-Intubation Checklist (30–40 s bedside checklist)	P—Position: Ear-to-sternal-notch alignment (maximizes glottic view, optimizes FRC). E—Equipment: DL/VL (if available), bougie, SGA, suction, vasopressor prepared. A—Airway Plan: Verbalize Plan A→B→C→D. R—Rescue: Confirm SGA size; scalpel–bougie FONA set visible. L—Lines/Vitals: BP cycling, oxygen source, waveform capnography (if available).	Reduces omission errors Reduces peri-intubation physiologic complications by 30–40% in ICU audits; improves completeness and reduces omission errors
Airway Briefing Card (laminated A5 card on trolley)	Plan A–D verbalized Attempt limit defined.	Improves shared mental model
Oxygenation Failure Trigger Card (monitor sticker or cart-mounted prompt)	Alerts team: Stop attempts if SpO_2_ < 90% or drop >5%. If oxygenation is not restored in 10–15 s → place SGA. Prioritizes oxygenation over intubation attempts.	Directly addresses the primary cause of morbidity in NAP4: prolonged laryngoscopy during falling SpO_2_. Trigger prompts shown to prevent fixation errors and improve cognitive offloading.
CICO Micro-Checklist (placed inside FONA tray lid)	Simple scalpel–bougie–tube steps: Vertical incision Horizontal incision Bougie in Tube over bougie.	Proven to improve decision time and reduce hesitancy in emergency FONA; micro-checklists recommended in DAS and AIDAA guidance.
Shared Mental Model Script	Team alignment in 10–15 s	Reduces hierarchical delay
20-Second Laryngoscopy Timer Cue	“20 s oxygenation check”	Human-factors principles support timed prompts to reduce task fixation; NAP4 emphasizes time-linked deterioration leading to severe complications.

APC: anatomical–physiological–contextual; AIDAA: All India Difficult Airway Association; CRM: crisis resource management; CICO: cannot intubate, cannot oxygenate; DAS: Difficult Airway Society; DL: direct laryngoscopy; FONA: front-of-neck access; FRC—functional residual capacity; ICU: intensive care unit; NAP4: Fourth National Audit Project of the Royal College of Anaesthetists; SGA: supraglottic airway; VL: video laryngoscopy.

**Table 9:** Practical system-level interventions for low-resource airway safety.

Domain	Intervention	Expected Impact
Equipment reliability	Daily airway cart checks Sealed grab-and-go kits	Prevents catastrophic delays
Workflow integration	Checklist embedded in routine practice	Improves consistency
Team behavior	Explicit attempt limits Empowered stop–oxygenate call	Earlier escalation
Training systems	Low-dose, high-frequency drills	Better skill retention
Cognitive aids	Posters, cards, tray-mounted prompts	Reduces cognitive load
Quality improvement	Event debriefs Airway metrics tracking	Continuous system learning

## Data Availability

No new data were created or analyzed in this study.

## Abbreviations

The following abbreviations are used in this manuscript:

ICU	Intensive care unit
ED	Emergency department
FONA	Front-of-neck access
SGA	Supraglottic airway
HFNO	High-flow nasal oxygen
NIV	Non-invasive ventilation
CICO	Cannot intubate, cannot oxygenate
